# The effect of rifampin on the pharmacokinetics of famitinib in healthy subjects

**DOI:** 10.1007/s00280-022-04474-8

**Published:** 2022-09-15

**Authors:** Ting Li, Xin Li, Xin Jiang, Chenjing Wang, Feifei Sun, Yanping Liu, Pingping Lin, Ping Shi, Yao Fu, Xiaomeng Gao, Yanyan Zhang, Yu Cao

**Affiliations:** 1 Phase I Clinical Research Center, The Affiliated Hospital of Qingdao University, Qingdao, 266003 China; 2 Clinical Pharmacology Department, Jiangsu Hengrui Pharmaceuticals Co. Ltd., Jiangsu, China

**Keywords:** Famitinib, Rifampin, Drug-drug interaction, CYP3A4, Pharmacokinetics

## Abstract

**Background:**

Famitinib is an oral, small-molecule, multi-targeted tyrosine kinase inhibitor under clinical investigation for the treatment of solid tumors. As famitinib is metabolized mainly by cytochrome P450 3A4 (CYP3A4), the study was conducted to investigate the effect of potent CYP3A4 inducer rifampin on the pharmacokinetics of famitinb.

**Methods:**

This single-center, single-arm and fixed-sequence drug–drug interaction study enrolled 21healthy Chinese male subjects. Subjects received a single oral dose of famitinib 25 mg on days 1 and 16 and repeated administration of oral rifampin 600 mg once daily on days 10–23. Blood samples were collected and plasma concentrations of famitinib were measured by validated liquid chromatography-tandem mass spectrometry (LC–MS/MS) method. Pharmacokinetic parameters were calculated using noncompartmental analysis and safety was assessed.

**Results:**

In the presence of rifampin, the famitinib geometric mean maximum plasma concentration (*C*_max_) and area under the plasma concentration–time curve from time zero to infinity (AUC_0–*∞*_) decreased by 48% and 69%, respectively, and the mean elimination half-life was shortened from 33.9 to 18.2 h. The geometric mean ratio (GMR) of famitinib *C*_max_ and AUC_0–*∞*_ and their 90% CI were 0.52 (0.50, 0.54) and 0.31 (0.29, 0.33). Single dose of famitinib 25 mg was well tolerated and eight subjects (38.1%) reported treatment emergent adverse events, which were all grade 1–2 in severity.

**Conclusion:**

Co-administration of rifampin considerably reduces plasma concentration of famitinb due to CYP3A4 induction. Concomitant administration of famitinib and strong CYP3A4 inducers should be avoided, whereas when simultaneous use with inducers of CYP3A4, dose adjustment of famitinb is recommended.

**Clinical trial registration number:**

NCT04494659 (July 31, 2020).

**Supplementary Information:**

The online version contains supplementary material available at 10.1007/s00280-022-04474-8.

## Introduction

Tyrosine kinase receptors (TKRs) are a family of cell surface growth factor receptors that play an essential role in the molecular pathways of cell survival and differentiation. Genetic alterations of TKRs may lead to tumorigenesis, progression, invasion and metastasis of malignant tumors [1]. Consequently, tyrosine kinase inhibitors (TKIs) against TKRs are designed for the treatment of multiple malignancies.

Famitinib malate (i.e. famitinib, SHR1020) is an orally active, small-molecule, multi-targeted and potent TKI that inhibits various TKRs including c-Kit, platelet-derived growth factor receptor (PDGFR), vascular endothelial growth factor receptor (VEGFR), FMS-like tyrosine kinase-3 receptor (FLT3) and RE arranged during Transfection (RET). Famitinib not only inhibits the angiogenesis, but also directly inhibits the expansion of tumor cells, which have been verified both in vivo and in vitro [2, 3]. Completed Phase I and II studies in patients with advanced solid cancer have demonstrated that famitinib is well tolerated and has a broad spectrum of antitumor activities [4–8]. Moreover, a series of phase II and III clinical trials (ClinicalTrials.gov Identifier: NCT04619433, NCT04335006, NCT03134872, NCT03764293, NCT03813784, etc.) are currently still ongoing to further evaluate the safety and efficacy of famitinib in non-small cell lung cancer (NSCLC), breast cancer, renal cell carcinoma (RCC), gastrointestinal stromal tumor (GIST) and many other solid tumors.

Famitinib is a structural analogue of sunitinib (Sutent^®,^ Pfizer). Compared with sunitinib, famitinib exhibits superior inhibition activities against multiple TKRs on various cell lines, and animal studies also demonstrated the higher potency of famitinib in tumor growth inhibition in human tumor xenograft models (unpubl. data). Moreover, clinical trials showed the similarity of the antitumor effects between famitinib and sunitinib, while famitinib could achieve the same efficacy with a lower therapeutic dose compared with sunitinib [2, 9].

Famitinib is slowly absorbed from the gut, extensively metabolized in the liver and eliminated mainly via biliary-fecal route (approximately 56.3% of the administered dose). Unchanged famitinib is the major circulating material [9] and the pharmacokinetics of famitinib in human has been assessed by Zhou et al. [8]. After a single oral administration, the peak concentrations (*C*_max_) of 4–27 mg famitinib occur within 3.3–5.3 h in cancer patients, while the mean time to maximum concentration (*T*_max_) of 25 mg famitinib is 5.9 ± 3.8 h in healthy subjects. There is no significant accumulation of famitinib. After continuously dosing, the accumulation ratios (Day28/Day1) of *C*_max_ and area under concentration time curve from zero to 24 h (AUC_0–24 h_) of famitinib are 2.15 and 2.53, respectively. Food demonstrates a minimal effect on the pharmacokinetics of famitinib [8].

In *vitro,* the metabolism of recombinant human isozymes and the microsomal chemical inhibition studies demonstrated that multiple enzymes, including cytochrome P450 (CYP) isoform CYP3A4/5, CYP1A1/2, aldehyde dehydrogenase and flavin mono-oxygenases 3, participated in the oxidative metabolism of famitinib. Of them, CYP3A4/5 was the key enzyme in *N*-desethylation and oxidative deamination to aldehyde, whereas CYP1A1/2 had the highest efficacy in oxidative defluorination and indolylidene hydroxylation. The metabolism studies showed that CYP3A4-mediated *N*-desethylation is most important to the famitinib metabolic clearance [9]. Therefore, a drug–drug interaction (DDI) between famitinib and inducers of CYP3A4 may occur.

Rifampicin (rifampin), one of the most effective and broad spectrum antimicrobials, is mainly used in the treatment of tuberculosis with a dosage of 600 mg once daily [10]. Rifampin induces a number of drug-metabolizing enzymes, while its major inducing effect is on CYP3A4. Induction occurs within 24 h of its administration, and full induction effect is reached in approximately 3 days after starting rifampin treatment [11]. Thus, rifampin is often used experimentally as a model compound to assess the effects of a strong CYP3A4 inducer [12].

To evaluate the influence of concomitant rifampin on the pharmacokinetics of famitinib, we conducted a single-center, single-arm and fixed-sequence study, the results of which provide insight into the effect of CYP3A4 on the bioavailability of famitinib.

## Materials and methods

The study was approved by the responsible ethics committee (Medical Ethics Committee of the Affiliated Hospital of Qingdao University, No.: QYFYEC 2018–055-01) and was performed at Phase I Clinical Research Center of the Affiliated Hospital of Qingdao University. The study protocol was conducted in accordance with the Declaration of Helsinki, Good Clinical Practice (GCP) and applicable laws and regulations of China National Medical Products Administration (NMPA). Written informed consent was obtained from all subjects before their participation in the study.

### Subjects

Sex-related differences have been proved to exist in the extent of CYP3A induction by rifampin [13] and age can also affect the clearance of certain CYP3A substrates [14]. To evaluate the effect of a strong CYP3A inducer on famitinib PK variability without the contribution of gender and age factors, the DDI study was conducted in healthy male adult subjects aged in 18–50 years, with a body mass index (BMI) between 19 and 28 kg/m^2^ and a minimum of 50 kg weight.

The main exclusion criteria included the following: history or presence of clinically significant systemic diseases or disorders; significant abnormalities in physical examination and essential laboratory tests; hypersensitivity to investigational products; use of any prescription medication or herbal preparations within 1 month, or use of over-the-counter medication or dietary supplements, within previous 2 weeks, especially drugs that induce or inhibit CYA3A enzyme; abuse of alcohol or drugs; smoking more than 5 cigarettes a day or urinalysis cotinine diagnosis being positive; consumption of any beverages or food containing caffeine or products rich in grapefruit, such as coffee, tea and chocolate, etc., within 48 h prior to dosing or during the trial.

The impact of cigarette smoking on famitinib is unclear now. Cigarette smoke exposure induces hepatic CYP enzymes, especially CYP1A1/2. Whereas CYP3A4 is identified as the principle isoform that mediates famitinib bioactivation in the intestine, CYP1A1/2 is found to be the primary enzyme responsible for famitinib bioactivation in the liver, kidney and lung [9]. Thus, the highly expressed CYP1A of smokers may elevate the risk of famitinib-induced hepatotoxicity and subjects enrolled in our study were all non-smokers, due to the high sensitivity of cotinine diagnosis.

### Study design

The DDI study was an open-label, self-contrast study comprising the following two periods: period 1 (Days 1–9), administration of a single oral dose of famitinib 25 mg alone in the morning of study day 1; period 2 (Days 10–23), repeated administration of oral rifampin 600 mg once daily for 14 days in the morning of days 10–23, and co-administration of a single dose of famitinib 25 mg on Day 16. The famitinib was supplied as 25 mg capsules (Jiangsu Hengrui Pharmaceuticals Co., Ltd, Jiangsu Province, China; batch no.:200304NU; expiration date: March 2022), while rifampin as 300 mg capsules (Shenyang Hongqi Pharmaceutical Co., Ltd., Liaoning Province, China, batch no.:2001011; expiration date: December 2020). In the two periods, oral doses of famitinib were administered after an overnight fast (at least 10 h) with 240 mL room-temperature water. Subjects were forbidden to drink water within 1 h before and after taking famitinib, and the lunch and dinner were provided at 4 h and 8 h,, respectively, post-drug administration (Day 1, 16). In the second period, subjects received rifampin in the fasting state with 240 mL water and had regular meals 1 h after the dose. Cigarettes, caffeinated drinks and alcoholic products were prohibited during the whole trial period, and when the subjects stayed in the study center (Days 1–4, 10–23), the only food permitted was prepared and provided at predetermined times by the researchers.

### Blood sampling and analytical methods

3 mL venous blood samples were collected in lithium-heparin tubes pre-dose and at 1, 2, 3, 4, 5, 6, 8, 12, 24, 48, 72, 96,144 and 192 h after each famitinib dose on the pharmacokinetic profiling days. Samples were centrifuged within 1 h at 1500xg for 10 min at 4℃ to separate the plasma, which was divided into two aliquots (drug monitoring at least 800ul and backup) and stored at – 80 °Cuntil analysis. The whole process was operated in yellow light and avoided being exposed to light because the famitinib metabolite concentrations decreased significantly after light exposure [15], which is similar to sunitinib [16].

Plasma concentrations of famitinib were analysed using a validated liquid chromatography-tandem mass spectrometry (LC–MS/MS) method [9, 15] at Frontage Laboratories Co., Ltd (Shanghai, China). The calibration curve for famitinib covered ranges of 0.050-100 ng/ml. The low limit of qualification (LLOQ) was 0.050 ng/ml. The accuracy, expressed as relative error (RE), was within ± 4.2% and the precision, expressed as coefficient of variation (CV), was < 7.6%, which were conducted on quality control samples with low (0.15 ng/ml), medium (4 ng/ml) and high (75 ng/ml) concentration levels.

### Pharmacokinetic calculations

Pharmacokinetic (PK) parameters for famitinib in plasma were estimated by noncompartmental analysis using Phoenix WinNonlin (version 7.0, Pharsight Corporation, St Louis, MO, USA). The primary endpoints were the maximum plasma famitinib concentration (*C*_max_), the area under the plasma concentration–time curve from time 0 to the last measured time point (AUC_0–*t*_), and the area under the plasma concentration–time curve from time 0 to infinity (AUC_0–*∞*_). The secondary PK parameters included time to *C*_max_ (*T*_max_) and the elimination half-life (*T*_1/2_), the terminal exponential rate constant (*λ*_*z*_), the apparent total body clearance (CL/F). *C*_max_ and *T*_max_ were observed directly from the original concentration–time data. AUC_0–*t*_ was determined up to the last observed quantifiable concentration using the trapezoidal rule, and AUC_0–*∞*_ was the sum of AUC_0–*t*_ and the extrapolated AUC (AUC_ex*t*rap_), which was calculated based on the last measurable concentration dividing by *λ*_*z*_. *λ*_*z*_ is the slope determined by a linear regression analysis of the terminal phase of the concentration–time profile and *T*_1/2_ was obtained as 0.693/*λ*_*z*_.

### Safety and tolerability

Safety and tolerability were evaluated by monitoring vital signs, physical examination, laboratory tests, electrocardiogram (ECG) and adverse events (AEs). Vital signs, including body temperature, blood pressure (BP) and heart rate, were measured and ECG were performed during baseline period, before drug administration and at 4, 24, 48, 192 h after famitinib administration. Clinical laboratory tests were conducted at screening, day 9, 15, and prior to discharge. AEs, including all subjective symptoms reported by subjects and objective signs observed by clinical investigators, were collected after dosing throughout the study.

### Statistical analysis

All subjects who received the study drug and for whom at least one PK parameter data was available were included in the PK analysis sets. The safety analysis sets included subjects who received at least one dose of famitinib or rifampin. Statistical analysis was performed using SAS 9.4 (SAS Inc., Cary, NC, USA). Assuming an intrasubject standard deviation of log-transformed *C*_max_ of 0.3, the tolerance of 80%, and geometric mean ratio (GMR) of 0.2 for AUC of famitinib co-administrated with rifampin versus famitinib only, the 90% confidence intervals (CI) was estimated 0.164–0.244 based on a sample of 18 completing subjects. Considering a drop-off of 15%, 21 subjects were enrolled. The measurement data were expressed as mean ± standard deviation (SD), and categorical variables were described by the percentage. A liner mixed-effects model with log-transformed PK parameters as response was used to calculate the GMR and associated two-sided 90%CI for AUC and *C*_max_, after eliminating the data with AUC__%Ex*t*rap_ (AUC_ex*t*rap_/AUC) greater than 20%. If the 90%CI was contained within the bounds 0.80–1.25, it was inferred that there was no drug interaction [17].

Safety parameters of the safety analysis sets were summarized using descriptive statistics. AEs were graded according to Common Terminology Criteria for Adverse Events (CTCAE, version 5.0) and coded using Medical Dictionary for Regulatory Activities (MedDRA, version 19.0) System Organ Class (SOC) and Preferred Terms (PT).

## Results

A total of 75 subjects were screened and 21 healthy male subjects were enrolled. All subjects completed the study except one dropping out before co-administration due to a protocol deviation (strenuous exercise and muscle injury). 21 subjects were all Han nationality, with the median age of 28 (range 20, 44) years, the mean (± SD) height of 171.4 ± 5.2 cm, mean weight of 67.7 ± 9.0 kg, mean body mass index (BMI) of 23.0 ± 2.5.

### Pharmacokinetics

**Fig. 1:**
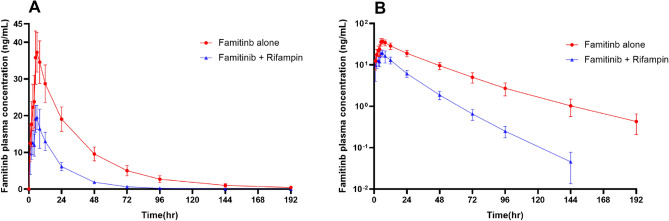
The mean (± SD) plasma concentration–time curves of famitinib after a single oral administration of 25 mg famitinib in the presence and absence of rifampin (**A** linear and **B** semi-logarithmic scale)

In the presence of rifampin, the geometric mean *C*_max,_ AUC_0–*t*_ and AUC_0–*∞*_ of famitinib decreased by 48%, from 38.7 to 20.1 ng/mL, 69% from 1298.5 to 405.0 h × ng/mL, and 69%, from 1320.5 to 407.4 h × ng/mL, respectively. The mean elimination *T*_1/2_ of famitinib when co-administered with rifampin compared with administered alone was shortened remarkably, 18.2 h and 33.9 h, respectively, while the median *T*_max_ was not affected, 5.5 h and 6.0 h, respectively. The GMR of famitinib *C*_max,_ AUC_0–*t*_ and AUC_0–*∞*_ and their 90% CI were 0.52 (0.50, 0.54), 0.31 (0.29, 0.33) and 0.31 (0.29, 0.33), respectively, which were blow the no DDI range of 0.80–1.25. Pharmacokinetic parameters during the two periods are listed in Tables 1 and 2 and the time profile of famitinib serum concentration is showed in Fig. 1.

**Table 1:** Pharmacokinetic parameters of famitinib in 21 Chinese healthy male subjects administered a single oral dose of famitinib 25 mg or after co-administration with steady-state rifampin

Pharmacokinetic parameter	Famitinib alone (*N* = 21)	Famitinib + Rifampin (*N* = 20^c^)
*C*_max_ (ng/mL)	38.7 (15.3)	20.1 (17.1)
AUC_0–*t*_ (h × ng/mL)	1298.5 (17.2)	405.0 (16.7)
AUC_0–*∞*_ (h × ng/mL)	1320.5 (17.4)	407.4 (16.6)
*T*_max_ (h)^a^	6.0 (5.0, 8.0)	5.5 (1.0, 8.0)
*T*_1/2_ (h)^b^	33.9 (6.6)	18.2 (3.2)
*λ*_*z*_ (1/h)	0.021 (21.16)	0.039 (20.07)
CL/F (L/h)	18.9 (18.0)	61.4 (16.5)

Values are geometric mean (geometric CV, %) unless otherwise noted

^a^Median (min, max)

^b^Mean (SD)

^c^One patient withdrew before the second dose of famitinib

**Table 2:** Geometric mean ratios (GMR) for famitinib *C*_max_, AUC_0–*t*_ and AUC_0–*∞*_ with 90% confidence intervals (CI)with and without rifampin

Parameters	GMR	Intra-subject CV (%)	90% CI
*C*_max_(ng/mL)	0.52	7.54	0.50, 0.54
AUC_0–*t*_(h × ng/mL)	0.31	11.86	0.29, 0.33
AUC_0–*∞*_(h × ng/mL)	0.31	12.21	0.29, 0.33

### Safety

All the 21 subjects were included in the safety analysis sets, of which eight subjects (8/21, 38.1%) reported treatment emergent AEs (TEAEs). No serious or significant TEAEs were reported and all TEAEs were spontaneously recovered without specific intervention. The adverse events occurred during the study were hyperbilirubinaemia, hypertriglyceridemia, neutropenia, leucocytopenia, ST segment abnormal, hyperpotassaemia, ulnar nerve injury, myalgia, angular cheilitis, anemia. Except one case of hyperbilirubinaemia, one case of neutropenia and two cases of hypertriglyceridemia which were grade 2, the TEAEs were all grade 1 in severity. One treatment-related TEAE (1/21, 4.8%) was recorded during famitinib alone period (Days 1–9), four treatment-related TEAEs in three subjects (3/21, 14.3%) were recorded during daily doses of rifampin alone (Days 10–15), and five treatment-related TEAEs in four subjects (4/20, 20%) were recorded after famitinib in combination with multiple doses of rifampin (Days 16–23). All AEs after 25 mg famitinib with and without rifampin were summarized in Table 3. The mean vital sign measurements and laboratory examinations after administrations were similar to those at baseline, and there was no clinically significant change in the follow-up safety parameters when famitinib given alone compared with co-administration of famitinib and rifampin.

**Table 3:** Summary of treatment emergent adverse events (TEAEs) after 25 mg famitinib with and without rifampin in healthy male subjects

TEAE	Famitinib alone (*N* = 21)	Rifampin alone (*N* = 21)	Famitinb + Rifampin (*N* = 20)
Hyperbilirubinaemia	1	0	0
hypertriglyceridemia	0	0	2
neutropenia	0	2	1
leucocytopenia	0	1	0
ST segment abnormal	0	0	1
Hyperpotassaemia	0	0	1
Ulnar nerve injury	0	1	0
Myalgia	0	1	0
Angular cheilitis	0	1	0
Anemia	0	0	1

## Discussion

This single-center, open-label study conducted in healthy male subjects evaluated the effects of CYP3A4 inducer, multiple-dose rifampin, on the pharmacokinetic profile of famitinib. The results indicate a significant drug–drug interaction between rifampin and famitinib. Co-administration of 600 mg rifampin with 25 mg famitinib significantly decreased famitinib *C*_max_ by 48% and AUC_0–*∞*_ by 69% compared with famitinib alone, which is likely explained by an enhanced presystemic elimination of famitinib. The reduction in the *T*_1/2_ of famitinib caused by rifampin indicated that systemic clearance of famitinib was increased, resulting from CYP3A4 induction. The PK parameters of a single dose of 25 mg famitinib were consistent with the results of a phase I study conducted by Zhou et al*.* in healthy subjects under fasting condition [8].

Except as a strong CYP3A inducer, rifampin is also recognized as a substrate and inducer of permeability glycoprotein (P-gp), an important ATP-binding cassette transporter, upon multiple dosing [18]. In addition, rifampin also displays acute inhibitory effects on hepatic uptake transporters (organic anion transporter family, OATP). Therefore, the interaction between famitinib and P-gp as well as OATP needs to be considered, to evaluate whether the changes of famitinib exposure caused by the strong CYP3A inducer are affected. The in vitro study shows that famitinib is neither a substrate, an inducer or inhibitor of P-gp in Caco-2 cells, nor a substrate of OATP, indicating the CYP enzyme induction is not incorrect assessment in our study.

As structural analogue, sunitinib is also mediated primarily by the CYP3A4 isozyme. CYP3A4 metabolizes sunitinib into an active metabolite, approximately 55% of the parent drug, which is further metabolized by CYP3A4 into inactive moieties [19]. Coadministration of sunitinib and rifampin was associated with a 4.8- and 2.3-fold reduction in sunitinib AUC and *C*_max_, while a 1.3- and 2.4 fold increase in terms of the active metabolite AUC and *C*_max_, compared with dosing healthy volunteers with sunitinib alone [20]. However, for famitinib, much lower exposure of *N*-Desethylfaminitib (M3) was observed, which is pharmacodynamically active but exhibits only 4.5% of parent drug [9]. Thus, the CYP3A-based drug–drug interaction of famitinib was less significant than that of sunitinib. Besides famitinib and its analogue sunitinib, rifampin can decrease exposure of other TKIs. *C*_max_ and AUC of imitinib, gefitinib, erlotinib and afatinib decreased by 54% and 74% [21], 65% and 83% [22], 39% and 69% [23], 22% and 34% [24], respectively, when simultaneous administrated with rifampin. Thus, when prescribed with these TKIs in clinical practice, the CYP3A4-inducing drugs need to be avoided and the alternative concomitant treatment is recommended for treating concurrent illness. If CYP3A4 inducers are necessary, the influence of strong CYP3A4 inducer-induced changes in drug exposure should be considered and the starting dose should be adjusted to provide adequate pharmacokinetic exposure, ensuring the antitumor effect. It is recommended that the sunitinib dose be increased to 175% of the recommended dose in patients who require concomitant use of a CYP3A4 inducer [25]. For famitinb, the dose adjustment guidelines are not yet available and warrant further studies.

Single doses of famitinib 25 mg were well tolerated in healthy subjects when given alone and in combination with multiple doses of rifampin. The major adverse reactions (incidence rate > 30%) after continuous administration of famitinib in cancer patients were hypertension, hand-food skin reaction, oral mucositis, bone marrow suppression, diarrhea, proteinuria, hypertriglyceridemia, fatigue, liver dysfunction, etc. [3, 5, 6, 8]. In this study, the famitinib-related adverse events were in line with the known safety profile. Food and Drug Administration (FDA) published a press announcement to be aware of nitrosamine impurities, some of which are classified as probable human carcinogens, in rifampin and restrict the use of rifampin on August 26, 2020. Unfortunately, all the subjects in the study had completed the administration before August 13, 2020. However, the carcinogenic potential of nitrosamine is closely related to lifetime exposure, and no related adverse events were observed with the much low dose of rifampin administered in our study. Nevertheless, the safety of rifampin in healthy volunteers deserves attention in future studies.

We acknowledge a number of limitations in our study. First, the study was conducted in healthy young male Chinese subjects. It is generally recognized that sex, age and ethnic differences may influence drug disposition. However, the DDI exploratory study with a small sample size is not suitable for the analysis of exposure differences between different factors. Second, the study focused on the effects of CYP3A4 inducer on the pharmacokinetics of single dose famitinib. The rifampin induction effects on steady-state famitnib in cancer patients would be more valuable to speculate on a solution to precisely dose famitinib. However, our results are necessary for future study design. Finally, only the unchanged famitinib was detected, not the metabolites. Quantifying the metabolite would help better explain the drug–drug interactions. However, the major metabolite M3 represents approximately 4.5% of the parent drug and PK analysis is not required for those whose metabolites exposure is less than 10%, according to the regulations of NMPA.

In conclusion, this study shows that pretreatment with rifampin considerably reduces plasma concentration of famitinb in healthy subjects due to CYP3A4 induction. Although the interaction of rifampin combined with famitinib in cancer patients in not clear, it is reasonable to assume that concomitant therapy with potent CYP3A4 inducer such as rifamipin may impair the therapeutic properties of famitinib in the treatment of malignant tumors. Concomitant administration of famitinib and strong CYP3A4 inducers should be avoided; when simultaneous use with inducers of CYP3A4 is necessary, dose adjustment of famitinb is, therefore, recommended.

## Supplementary Information

Below is the link to the electronic supplementary material.

Supplementary file1 (DOCX 34 KB)

## Data Availability

The data that support the findings of this study are available on request from the corresponding author. The data are not publicly available due to privacy or ethical restrictions.
