# Supplementary material for: The effect of rifampin on the pharmacokinetics of famitinib in healthy subjects
Source: Cancer Chemother Pharmacol. 2022 Sep 15;90(5):409–15. doi: 10.1007/s00280-022-04474-8 (PMC9556364; doi:10.1007/s00280-022-04474-8)
Supplement: Supplementary file 1 — Supplementary file1 (DOCX 34 KB) [file 280_2022_4474_MOESM1_ESM.docx]

**S Table 1. Summary description of plasma concentrations of famitinib--famitinib alone period**

|  | **Plasma concentration of famitinib corresponding to each sampling time point (ng / mL)** | | | | | | | |
| --- | --- | --- | --- | --- | --- | --- | --- | --- |
| **Subject** | **Before dosing** | **1h** | **2h** | **3h** | **4h** | **5h** | **6h** | **8h** |
|  | | | | | | | | |
| 1 | BLQ<0.050 | 12.773 | 17.877 | 20.490 | 20.547 | 32.913 | 36.103 | 28.312 |
| 2 | BLQ<0.050 | 10.295 | 15.400 | 16.692 | 21.652 | 34.455 | 36.767 | 40.047 |
| 3 | BLQ<0.050 | 10.969 | 16.503 | 20.933 | 19.902 | 38.570 | 33.489 | 35.942 |
| 4 | BLQ<0.050 | 13.903 | 18.719 | 28.240 | 26.805 | 47.417 | 38.089 | 34.604 |
| 5 | BLQ<0.050 | 26.555 | 35.638 | 37.318 | 37.143 | 48.115 | 45.213 | 44.525 |
| 6 | BLQ<0.050 | 12.172 | 19.290 | 24.870 | 28.028 | 34.268 | 44.283 | 42.144 |
| 7 | BLQ<0.050 | 10.070 | 11.263 | 24.032 | 28.292 | 39.992 | 43.837 | 36.756 |
| 8 | BLQ<0.050 | 23.342 | 27.171 | 35.652 | 40.156 | 39.262 | 41.218 | 37.274 |
| 9 | BLQ<0.050 | 11.725 | 17.301 | 17.196 | 20.043 | 34.731 | 38.122 | 37.931 |
| 10 | BLQ<0.050 | 7.749 | 11.179 | 13.891 | 16.081 | 23.153 | 30.476 | 31.453 |
| 11 | BLQ<0.050 | 13.952 | 19.862 | 21.638 | 19.854 | 38.641 | 37.003 | 34.655 |
| 12 | BLQ<0.050 | 8.453 | 16.456 | 20.909 | 21.159 | 37.246 | 33.261 | 28.227 |
| 13 | BLQ<0.050 | 16.495 | 17.403 | 26.523 | 24.187 | 34.840 | 32.099 | 26.193 |
| 14 | BLQ<0.050 | 11.559 | 19.212 | 20.328 | 24.994 | 39.863 | 44.461 | 38.443 |
| 15 | BLQ<0.050 | 9.713 | 16.738 | 19.378 | 22.750 | 36.023 | 40.451 | 33.433 |
| 16 | BLQ<0.050 | 14.845 | 25.161 | 25.658 | 36.134 | 38.105 | 44.411 | 40.722 |
| 17 | BLQ<0.050 | 9.794 | 11.223 | 13.587 | 11.368 | 23.878 | 30.717 | 30.892 |
| 18 | BLQ<0.050 | 13.641 | 13.668 | 23.069 | 18.316 | 27.444 | 31.790 | 27.531 |
| 19 | BLQ<0.050 | 7.456 | 15.742 | 22.375 | 23.577 | 47.892 | 38.642 | 41.552 |
| 20 | BLQ<0.050 | 5.075 | 7.198 | 13.449 | 15.599 | 23.899 | 27.121 | 23.131 |
| 21 | BLQ<0.050 | 10.847 | 19.362 | 21.709 | 23.040 | 31.460 | 34.485 | 32.006 |
|  |  |  |  |  |  |  |  |  |
|  | **Plasma concentration of famitinib corresponding to each sampling time point (ng / mL)** | | | | | | | |
| **Screening number** | **12h** | **24h** | **48h** | **72h** | **96h** | **144h** | **192h** |  |
|  | | | | | | | | |
| 1 | 22.545 | 13.952 | 7.257 | 3.686 | 2.365 | 0.893 | 0.339 |  |
| 2 | 33.868 | 22.869 | 9.513 | 4.390 | 2.387 | 0.785 | 0.310 |  |
| 3 | 32.063 | 18.719 | 8.254 | 3.971 | 1.873 | 0.595 | 0.302 |  |
| 4 | 29.892 | 18.827 | 8.105 | 3.582 | 1.665 | 0.455 | 0.177 |  |
| 5 | 36.325 | 23.343 | 11.504 | 6.835 | 3.753 | 1.139 | 0.295 |  |
| 6 | 38.189 | 22.964 | 11.192 | 5.985 | 3.141 | 1.206 | 0.575 |  |
| 7 | 26.846 | 18.391 | 9.732 | 5.799 | 2.824 | 1.138 | 0.524 |  |
| 8 | 30.046 | 23.365 | 11.197 | 6.271 | 3.335 | 1.141 | 0.462 |  |
| 9 | 32.411 | 22.823 | 12.102 | 7.017 | 4.484 | 1.626 | 0.706 |  |
| 10 | 29.990 | 20.812 | 11.799 | 6.466 | 3.258 | 1.491 | 0.718 |  |
| 11 | 28.697 | 16.996 | 7.958 | 4.181 | 2.306 | 0.727 | 0.307 |  |
| 12 | 22.626 | 13.694 | 6.357 | 2.677 | 1.285 | 0.432 | 0.155 |  |
| 13 | 21.253 | 14.316 | 6.794 | 3.396 | 1.650 | 0.652 | 0.242 |  |
| 14 | 29.809 | 17.298 | 7.622 | 3.152 | 1.224 | 0.295 | 0.098 |  |
| 15 | 27.794 | 17.947 | 7.660 | 3.579 | 1.587 | 0.483 | 0.177 |  |
| 16 | 35.209 | 23.155 | 12.046 | 6.840 | 3.633 | 1.580 | 0.650 |  |
| 17 | 22.673 | 17.159 | 10.239 | 6.734 | 3.718 | 1.789 | 0.830 |  |
| 18 | 25.658 | 16.424 | 9.122 | 4.933 | 2.477 | 0.934 | 0.363 |  |
| 19 | 31.489 | 22.872 | 12.117 | 5.444 | 3.253 | 1.050 | 0.410 |  |
| 20 | 20.180 | 15.646 | 10.002 | 5.401 | 3.695 | 1.762 | 0.781 |  |
| 21 | 25.649 | 18.793 | 10.356 | 5.502 | 3.276 | 1.408 | 0.642 |  |
|  |  |  |  |  |  |  |  |  |

**S Table 2. Summary description of plasma concentrations of famitinib--famitinib + rifampin period**

|  | **Plasma concentration of famitinib corresponding to each sampling time point (ng / mL)** | | | | | | | |
| --- | --- | --- | --- | --- | --- | --- | --- | --- |
| **Screening number** | **Before dosing** | **1h** | **2h** | **3h** | **4h** | **5h** | **6h** | **8h** |
|  | | | | | | | | |
| 1 | BLQ<0.050 | 10.712 | 13.531 | 12.982 | 8.742 | 16.668 | 15.506 | 13.607 |
| 2 | BLQ<0.050 | 10.116 | 14.964 | 13.901 | 14.747 | 25.975 | 24.692 | 24.906 |
| 3 | BLQ<0.050 | 10.765 | 16.225 | 11.282 | 10.966 | 20.293 | 19.501 | 17.258 |
| 4 | BLQ<0.050 | 19.718 | 17.565 | 16.888 | 16.887 | 21.859 | 19.687 | 19.760 |
| 5 | BLQ<0.050 | 23.441 | 16.778 | 17.548 | 14.176 | 21.043 | 21.937 | 21.381 |
| 6 | BLQ<0.050 | 7.776 | 14.862 | 15.259 | 13.324 | 18.925 | 21.972 | 22.088 |
| 7 | BLQ<0.050 | - | - | - | - | - | - | - |
| 8 | BLQ<0.050 | 10.518 | 14.759 | 13.022 | 12.820 | 19.573 | 21.751 | 17.194 |
| 9 | BLQ<0.050 | 3.966 | 13.699 | 15.073 | 13.884 | 16.893 | 20.560 | 18.589 |
| 10 | BLQ<0.050 | 5.914 | 7.465 | 8.838 | 9.347 | 12.716 | 13.295 | 14.154 |
| 11 | BLQ<0.050 | 0.552 | 3.285 | 7.065 | 11.443 | 20.885 | 23.440 | 20.082 |
| 12 | BLQ<0.050 | 12.500 | 15.622 | 13.314 | 17.240 | 17.053 | 19.179 | 15.200 |
| 13 | BLQ<0.050 | 4.647 | 8.853 | 12.114 | 13.801 | 17.122 | 21.570 | 18.343 |
| 14 | BLQ<0.050 | 15.609 | 13.667 | 12.313 | 12.155 | 19.816 | 19.751 | 15.005 |
| 15 | BLQ<0.050 | 11.994 | 16.315 | 14.027 | 13.689 | 21.770 | 21.278 | 15.815 |
| 16 | BLQ<0.050 | 14.611 | 19.826 | 17.332 | 13.608 | 22.538 | 21.342 | 17.957 |
| 17 | BLQ<0.050 | 1.812 | 3.411 | 5.397 | 5.156 | 14.295 | 14.830 | 11.990 |
| 18 | BLQ<0.050 | 8.640 | 8.191 | 8.789 | 10.280 | 18.207 | 15.025 | 12.882 |
| 19 | BLQ<0.050 | 6.535 | 14.774 | 12.359 | 8.544 | 26.542 | 23.245 | 23.009 |
| 20 | BLQ<0.050 | 4.958 | 9.501 | 10.523 | 10.239 | 14.625 | 15.367 | 13.079 |
| 21 | BLQ<0.050 | 9.293 | 9.987 | 14.634 | 8.814 | 15.665 | 16.932 | 14.206 |
|  |  |  |  |  |  |  |  |  |
|  | **Plasma concentration of famitinib corresponding to each sampling time point (ng / mL)** | | | | | | | |
| **Screening number** | **12h** | **24h** | **48h** | **72h** | **96h** | **144h** | **192h** |  |
|  | | | | | | | | |
| 1 | 10.125 | 5.201 | 1.523 | 0.642 | 0.267 | 0.063 | BLQ<0.050 |  |
| 2 | 18.760 | 8.717 | 2.168 | 0.667 | 0.261 | 0.055 | BLQ<0.050 |  |
| 3 | 11.854 | 4.514 | 1.255 | 0.311 | 0.122 | BLQ<0.050 | BLQ<0.050 |  |
| 4 | 13.476 | 6.771 | 1.960 | 0.602 | 0.206 | 0.057 | BLQ<0.050 |  |
| 5 | 16.479 | 7.192 | 1.981 | 0.641 | 0.249 | BLQ<0.050 | BLQ<0.050 |  |
| 6 | 15.079 | 7.459 | 2.284 | 0.822 | 0.328 | 0.070 | BLQ<0.050 |  |
| 7 | - | - | - | - | - | - | - |  |
| 8 | 12.851 | 6.286 | 1.725 | 0.539 | 0.206 | 0.053 | BLQ<0.050 |  |
| 9 | 14.643 | 6.807 | 2.061 | 0.733 | 0.240 | BLQ<0.050 | BLQ<0.050 |  |
| 10 | 11.152 | 5.753 | 2.144 | 0.767 | 0.269 | 0.072 | BLQ<0.050 |  |
| 11 | 14.007 | 6.065 | 1.686 | 0.537 | 0.237 | 0.050 | BLQ<0.050 |  |
| 12 | 9.937 | 5.532 | 1.429 | 0.432 | 0.168 | BLQ<0.050 | BLQ<0.050 |  |
| 13 | 14.329 | 7.040 | 2.362 | 0.852 | 0.290 | 0.063 | BLQ<0.050 |  |
| 14 | 10.247 | 4.040 | 0.918 | 0.220 | 0.089 | BLQ<0.050 | BLQ<0.050 |  |
| 15 | 12.360 | 5.593 | 1.614 | 0.591 | 0.185 | BLQ<0.050 | BLQ<0.050 |  |
| 16 | 13.791 | 6.278 | 2.149 | 0.800 | 0.302 | 0.065 | BLQ<0.050 |  |
| 17 | 9.364 | 4.988 | 1.809 | 0.792 | 0.345 | 0.082 | BLQ<0.050 |  |
| 18 | 10.898 | 4.842 | 1.570 | 0.514 | 0.219 | 0.051 | BLQ<0.050 |  |
| 19 | 16.015 | 7.070 | 2.769 | 1.047 | 0.407 | 0.083 | BLQ<0.050 |  |
| 20 | 11.441 | 5.582 | 1.812 | 0.712 | 0.290 | 0.064 | BLQ<0.050 |  |
| 21 | 13.329 | 6.911 | 2.173 | 0.823 | 0.325 | 0.082 | BLQ<0.050 |  |
|  |  |  |  |  |  |  |  |  |

Note: for the concentration data whose concentration is lower than LLOQ, the list is uniformly calculated by " BLQ<0.050", and is uniformly calculated with "0" when describing and counting.
